# Rho Kinase Pathway Alterations in the Brain and Leukocytes in Huntington’s Disease

**DOI:** 10.1007/s12035-015-9147-9

**Published:** 2015-05-05

**Authors:** K. Lakshmi Narayanan, Vanita Chopra, H. Diana Rosas, Keith Malarick, Steven Hersch

**Affiliations:** Department of Neurology, MassGeneral Institute for Neurodegenerative Disease, Harvard Medical School, Massachusetts General Hospital, East, Bldg 114, Room 2005, Charlestown, MA 02129-4404 USA; Athinoula A. Martinos Center for Biomedical Imaging, Massachusetts General Hospital, Charlestown, MA 02129 USA; Center for Neuroimaging of Aging and Neurodegenerative Diseases, Massachusetts General Hospital, Charlestown, MA 02129 USA

**Keywords:** Huntington’s disease, Rho kinase pathway, mRNA expression, Biomarker

## Abstract

Huntington’s disease (HD) is a fatal neurodegenerative disease caused by an expanded polyglutamine tract in the huntingtin gene. Therapeutic approaches targeting mutant huntingtin (mtHtt) or its downstream toxic consequences are under development, including Rho kinase pathway inhibition. We investigated the messenger RNA (mRNA) expression of Rho kinase pathway genes, including RhoA (Ras homolog family member A), ROCK1 (Rho-associated kinase1), PRK2 (protein kinase C-related protein kinase 2), Profilin1, cofilin1, MYPT1 (myosin phosphatase target subunit 1), and LIMK1 (LIM domain kinase 1) in HD human blood leukocytes, postmortem brain, and in R6/2 HD mouse brain tissue using qPCR. RhoA, ROCK1, PRK2, Profilin1, cofilin1, and MYPT1 were significantly increased in HD blood compared to controls. In frontal cortex of HD postmortem brain tissue, the expression of RhoA, ROCK1, PRK2, Profilin1, and MYPT1 were also significantly increased. In the brain from 4-week-old R6/2 mice, the expression of Rock1, Prk2, Cofilin1, and MYPT1 was significantly increased while RhoA, Rock1, Profilin1, Cofilin1, and Mypt1 were increased and Limk1 mRNA decreased in 13-week-old R6/2 mice. Western blot analysis using human postmortem tissues for ROCK1 and Profilin1 demonstrated significantly increased protein levels, which correlated with the mRNA increases. Collectively, we have shown the panel of Rho kinase pathway genes to be highly altered in human HD blood, postmortem brain tissue, and in R6/2 mice. These studies confirm that HD upregulates the Rho kinase pathway and identifies mRNAs that could serve as peripheral markers in HD patients and translational markers in HD mouse models.

## Introduction

Huntington’s disease (HD) is a fatal, autosomal dominant disorder caused by a pathologic expansion of CAG repeats in the IT15 gene, which encodes the ubiquitously expressed huntingtin protein [1]. HD is characterized clinically by progressive decline in motor, cognitive, and behavioral function due to selective neurodegeneration that is especially prominent in the striatum and cortex [2]. While there are not yet any disease modifying treatments that have been demonstrated to delay or slow HD in patients, a number of potential targets have been identified and validated preclinically in HD models. One such promising target is inhibition of the Rho kinase pathway, which reduces mtHtt aggregation, enhances huntingtin clearance, and improves motor function in the R6/2 transgenic model of HD [3, 4], perhaps by modulating p21-activated kinase1 (Pak1), a downstream target protein of the Rho family that enhances mtHtt aggregation in cell culture and colocalizes with mtHtt in postmortem HD brain [5]. The Rho-associated kinase (ROCK1) inhibitor Y27632 emerged from an in vitro screen for molecules that reduce polyglutamine toxicity and subsequently was shown to reduce huntingtin toxicity and aggregation in cellular and drosophila models of HD [6, 7] and to enhance both proteasomal degradation of Htt and macroautophagy [8, 9]. In genome-wide microarray analyses of altered gene expression in leukocytes from HD patients, we identified two Rho kinase pathway messenger RNAs (mRNAs) amongst those most significantly elevated in HD; ROCK1 [10] and Cofilin1 [11] suggesting involvement of the pathway outside of the CNS. To assess the potential of Rho kinase genes as potential peripheral markers for HD in human or as pharmacodynamics markers to enable developing treatments targeting the Rho kinase pathway, we used quantitative real-time PCR of RhoA, ROCK1, PRK2, Profilin1, cofilin1, MYPT1, and LIMK1 to examine the pathway in more detail in leukocytes from HD patients as well as in human postmortem and R6/2 transgenic mouse brain tissue.

## Materials and Methods

### Antibodies and Reagents

Rock1 antibody (cat#4035, Cell Signaling), Profilin1 (cat#MABT500, Millipore), Cofilin1 (cat#MABT510, Millipore), beta-actin antibody (Cat#A2066, Sigma), and anti-rabbit IgG antibody conjugated to horse radish peroxidase were purchased from Abcam. Polyvinyldifluoride (PVDF) membrane (Bio-Rad) and the enhanced chemiluminescence kit were from Perkin Elmer. All other chemicals were from Sigma (St. Louis, USA).

### Animals

R6/2 transgenic HD mice were obtained from Jackson Laboratories (Bar Harbor, ME). R6/2 mice used in the study were generated by backcrossing male R6/2 mice with C57BL/6 × CBA F1 female mice. Mice were genotyped by PCR using tail-tip DNA (CAG repeat length of 135–140). We used wild-type littermates as controls. Mice were housed with a 12-h light/dark cycle in a humidity- and temperature-controlled atmosphere with ad libitum access to food and water. Mice were euthanized at the end of 4 and 13 weeks of age. Brains were removed, and striatum from the brain were dissected, snap frozen, and used for the RNA extraction. All animal experiments were carried out in accordance with the National Institutes of Health guidelines for animal research and were approved by the MGH Animal Care and Use Committee.

### Human Blood and Postmortem Brain Tissues

#### Human Subjects

Banked RNA samples from HD and control subjects participating in the REVEAL-HD biomarker program at MGH were used for this study (Table 1). The symptomatic HD patients had unequivocal motor symptoms based on the Unified Huntington’s Disease Rating Scale (UHDRS) and a known trinucleotide repeat expansion. Age and gender-matched control subjects were volunteers with no significant illnesses, most of whom were family members of the HD subjects. Human postmortem brain tissues were obtained from the Tissue Resource Centre of the Alzheimer Disease Research Center (ADRC) at Massachusetts General Hospital and Neurological Foundation of New Zealand Human Brain Bank, Auckland, and were used for this study. Procedures were explained and consent was obtained according to the Declaration of Helsinski (BMJ 1991; 302: 1194). Study protocols were approved by the Partners Internal Review Board (IRB).

**Table 1:** Clinical characteristics of healthy patients and symptomatic HD patients

Samples	Number	Gender	Age	Mean age
Controls (*n* = 15)	7	Male	32–77	51.1 ± 15.3
	8	Female	46–61	52.6 ± 4.9
Symptomatic HD (*n* = 16)	8	Male	48–65	60.6 ± 5.6
	8	Female	46–67	55.2 ± 7.3

### RNA Extraction and cDNA Synthesis

#### RNA Extraction from Human Blood

Whole venous blood was collected directly into Paxgene collection tubes (Paxgene™ Blood RNA Tube, Hombrechtikon, CH) which contain an RNA-stabilizing additive. Paxgene tubes as show in Table 1 were processed as previously described [12]. RNA extractions were carried out using the automated QIAcube system as per the Paxgene procedure including DNAse treatment. RNA quality was assessed using the RNA 6000 NanoChip kit and an Agilent 2100 Bioanalyzer (Agilent Technologies, USA) and spectrophotometry. Complementary DNA (cDNA) was synthesized using iScript cDNA synthesis kit (Bio-Rad) as per the manufacturer’s protocol.

**Table 2:** Clinical characteristics of postmortem brain tissues of control and symptomatic HD

Samples	Gender	Age	Mean AGE	Postmortem delay (h)
Controls (*n* = 14)	Male and female	41–78	56.3 ± 12.4	7–23
HD (*n* = 14)	Male and female	35–74	52.9 ± 10.9	4–19

#### RNA Extraction from Postmortem Human Brain and R6/2 Mouse Brain

Total RNA was isolated from 250 to 300 mg of the frontal cortex from postmortem human brain tissue slices obtained from the Tissue Resource Centre of the Alzheimer Disease Research center (ADRC) at Massachusetts General Hospital and R6/2 and control mouse striatum with 2 ml of TRIZOL reagent (Invitrogen, Carlsbad, CA, USA) (Table 2). The tissue was homogenized and RNA extraction was carried out following the manufacturer’s protocol. Two micrograms of total RNA were used for the reverse transcription following the DNase elimination procedure. cDNA was synthesized using iScript cDNA synthesis kit (Bio-Rad).

### Real-Time PCR

Briefly, for each gene of interest, primers were designed using the NCBI primer pick program and the primers were tested using the relative standard curve method. We used the CFX96 real-time PCR detection system (Bio-Rad). All reactions were performed in a 15-μl total volume that contained 2.5 μl of cDNA (1:10 dilution of reverse transcribed samples) and 12.5 μl of PCR reaction mixture containing specific primers (QuantiTech SYBR Green PCR kit, Qiagen). The primers used for the human samples and mouse samples are shown in Tables 3 and 4, respectively. The amplification consisted of an initial denaturation cycle at 95 °C for 15 min, followed by 45 cycles of 30 s at 94 °C, 30 s at 60 °C, and 30 s at 72 °C. Specific product formation was confirmed by melting curve analysis (55–95 °C).

**Table 3:** Sequences of the PCR primers for human samples

Gene	Orientation	Sequence (5′–>3′)	Size (in bp)
RhoA	Forward	TGAGCACACAAGGCGGGAGC	105
	Reverse	CACTCCATGTACCCAAAAGCGCCA	
ROCK1	Forward	GTCTGAGCAGTTGGCGCGAGG	143
	Reverse	GCATGCTGTTTGCTTCTTCAAGCCG	
PRK2	Forward	GCCCTCAAGCTCCTGTGCCTAC	128
	Reverse	GGCTGGAGGAGGTTCAGGCTCA	
Profilin1	Forward	GCCGGGTGGAACGCCTACAT	120
	Reverse	GACGAACGTTTTCCCGGGGACG	
Cofilin1	Forward	GGCGGTGCTCTTCTGCCTGA	110
	Reverse	AGGTGGCGTAGGGGTCGTCG	
MYPT1	Forward	GGACGCGAAGCAGAAGCGGA	119
	Reverse	AGACGGCGCCATCGTCGAAC	
LIMK1	Forward	GTGCGAGATCATCGGGCGGG	143
	Reverse	AACAGCGCACGGTGATGGGG	
Beta-actin	Forward	GGACACCCCACGCCAGTTCG	133
	Reverse	ACCGTGCGATCCCCATTGGC

**Table 4:** Sequences of PCR primers for mouse samples

Gene	Orientation	Sequence (5′–>3′)	Size (in bp)
mRhoA	Forward	CCGCCTGCGGCCTCTCTCTT	197
	Reverse	TGGCCAACTCCCGTCTCGTGT	
mRock1	Forward	AGAAAGAGGACTTGATTTCCCCGTGC	155
	Reverse	ACGGACAAAGCCAGATGGTGGG	
mPrk2	Forward	GTGCCAGTCGTTGACGCACG	112
	Reverse	CACGAGGTGGGGCTGGAGGA	
mProfilin1	Forward	CGTAGGCTACAAGGACTCGC	291
	Reverse	ACACCTTCTTTGCCCATCAG	
mCofilin1	Forward	CTGGGCCCCCGAGAATGCAC	136
	Reverse	GGTGCAGCGGTCCTTGACCT	
mMYPT1	Forward	AAGCGCTCCGTCGTCGTCCT	112
	Reverse	TCCCCGGGAGTAGGCAGAGGT	
mLimk1	Forward	CGGAATGTGCCGCTGGACGA	126
	Reverse	CAGGGGGCTGGGATCCGACA	
mBeta-actin	Forward	ACCGTGAAAAGATGACCCAG	273
	Reverse	TCTCAGCTGTGGTGGTGAAG	

### Western Blot Analysis

Briefly, R6/2 mouse brain tissues were homogenized in nine volumes of lysis buffer containing a protease inhibitor mixture. After incubation on ice for 30 min, lysates were briefly sonicated. Protein concentrations were determined using Bio-Rad DC protein assay reagent (Bio-Rad). Equal amounts of protein were boiled for 5 min in 6× SDS sample buffer and then separated by 7.5 % SDS-PAGE and electrophoretically transferred to a polyvinylidene difluoride membrane. The membranes were blocked in 5 % skim milk in 0.05 % tween 20 in Tris-buffered saline for 45 min and then incubated with Rock1 (1:150) primary antibody and beta-actin primary antibody (1:5000) overnight at 4 °C. The membranes were washed three times in TBS-T and incubated for 1 h with horseradish peroxidase-conjugated secondary antibody (1:2000). After the incubation period, the membranes were washed thrice in TBS-T. Immunoreactive proteins were detected using enhanced chemiluminescence (Perkin Elmer).

Postmortem human brain tissues obtained from the Neurological Foundation of New Zealand Human Brain Bank (Table 2) were homogenized in nine volumes of RIPA buffer containing a protease inhibitor mixture and tissues were incubated on ice for 10 min. Lysates were spun at 10,000 rpm for 10 min and supernatants were used for the protein estimation. Equal amounts of protein were boiled for 5 min and then samples were separated by 7.5 % SDS-PAGE gels for Rock1, 15 % SDS-PAGE gels for Profilin1 and cofilin1. The membrane were blocked in 5 % skim milk in 0.05 % tween 20 in Tris-buffered saline for 45 min and then incubated with primary antibodies for Rock1 (1:100), Profilin1 (1:5000), Cofilin1 (1:1000), or beta-actin (1:10,000) overnight at 4 °C. The membranes were washed three times in TBS-T and incubated for 1 h with horseradish peroxidase conjugated secondary antibody (1:2000). After the incubation period, the membranes were washed thrice in TBS-T. Immunoreactive proteins were detected using enhanced chemiluminescence.

### Statistical Analysis

Values are expressed as mean ± standard error of the mean. All the qPCR experiments were performed in quadruplicate. Statistical analyses were performed using GraphPad Prism version 5.0 (GraphPad Software, San Diego, USA) using Student’s *t* test (one-tailed). Results were considered statistically significant when *p* ˂ 0.05.

## Results

### Expression Levels of Rho Kinase Pathway Genes in the Cellular Blood of Patients with HD and in Frontal Cortex of Postmortem HD Brain

**Fig. 1:**
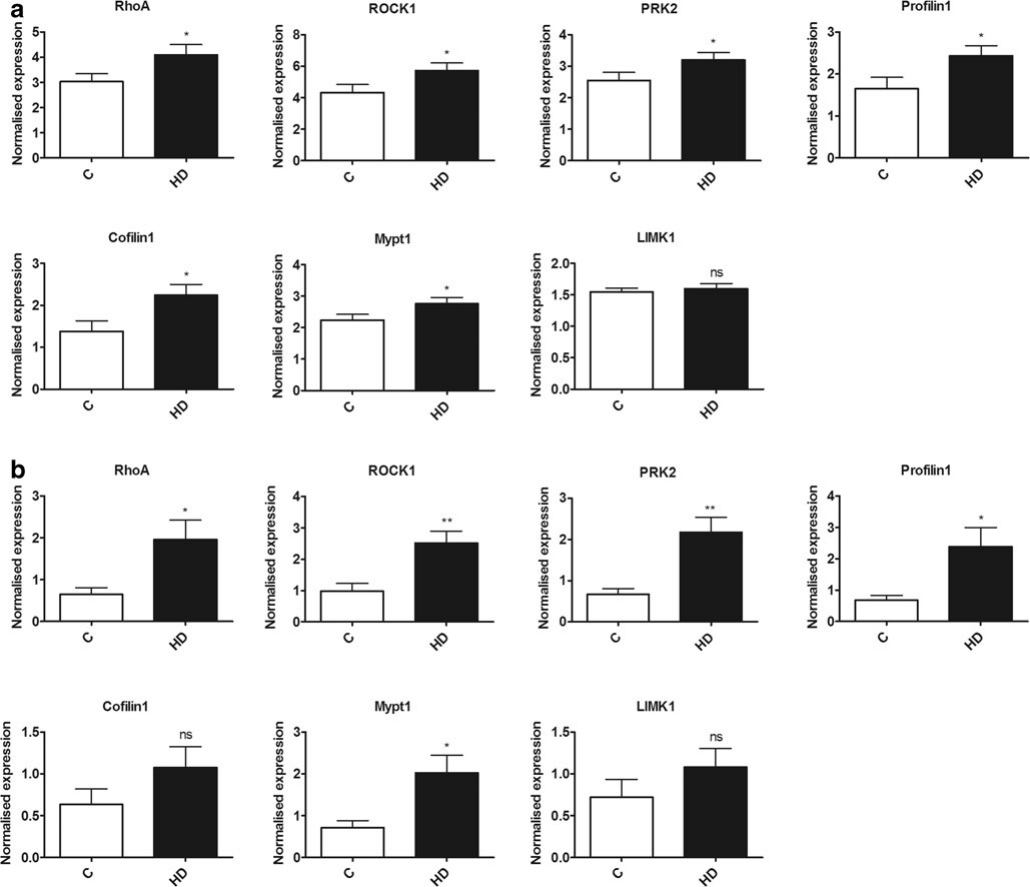
Expression levels of Rho kinase pathway genes in the cellular blood of patients with HD and in the frontal cortex of postmortem HD brain tissues quantified using qPCR. **a** Levels of RhoA, ROCK1, PRK2, Profilin1, Cofilin1, and MYPT1 mRNA were significantly increased in the cellular blood of patients with HD (*n* = 13–15) compared with age- and gender-matched controls (*n* = 12–14). LIMK1 mRNA levels were not altered. **b** Levels of RhoA, ROCK1, PRK2, Profilin1, and MYPT1 mRNA were significantly increased in postmortem brain of individuals with HD (*n* = 8–9) compared with controls (*n* = 5). There was no difference in expression levels of Cofilin1 or LIMK1. Expression was normalized with β-actin control. Data were expressed as mean ± S.E.M. *p ˂ 0.05, compared with the control

To determine whether Rho kinase pathway genes are altered in HD, we extracted RNA from age-matched symptomatic HD and healthy controls blood samples. cDNAs were synthesized and we carried out qPCR for the panel of genes in the Rho kinase pathway. We first conducted a preliminary study using a limited sample set (*n* = 5–8) and found increases in fold change at mRNA levels of Rho kinase pathway genes ranges from 1.274 to 1.636 (data not shown). Replication using an independent sample set with *n* = 13–15, revealed similar results: There were significant increases in the mRNA levels of RhoA (1.35-fold, *p* = 0.0289), ROCK1 (1.32-fold, *p* = 0.0296), PRK2 (1.26-fold, *p* = 0.0378), Profilin1 (1.47-fold, *p* = 0.0202), Cofilin1 (1.63-fold, *p* = 0.0129), and MYPT1 (1.23-fold, *p* = 0.0306) as shown in Fig. 1a, suggesting significant modulation of the Rho kinase pathway in human HD leukocytes. There was no significant change in mRNA levels of LIMK1 (*p* = 0.3119). To assess whether the Rho kinase pathway alterations in the blood are consistent with changes in HD brain, we extracted RNA from the frontal cortex of postmortem HD brain tissue and controls. cDNA was synthesized and we carried out qPCR for the panel of genes from the Rho kinase pathway. We found elevated mRNA levels of RhoA (3.02-fold, *p* = 0.0323), ROCK1 (2.58-fold, *p* = 0.0081), PRK2 (3.22-fold, *p* = 0.0049), Profilin1 (3.53-fold, *p* = 0.0267), MYPT1 (2.85-fold, *p* = 0.019) in postmortem brain of individuals with HD compared with controls (Fig. 1b), suggesting that Rho kinase pathway genes are similarly altered in the postmortem HD brain tissues. There was no significant difference in the expression levels of Cofilin1 (*p* = 0.1263) and LIMK1 (*p* = 0.15) in HD brain.

### Expression Levels of Rho Kinase Pathway Genes in Murine Model of HD

**Fig. 2:**
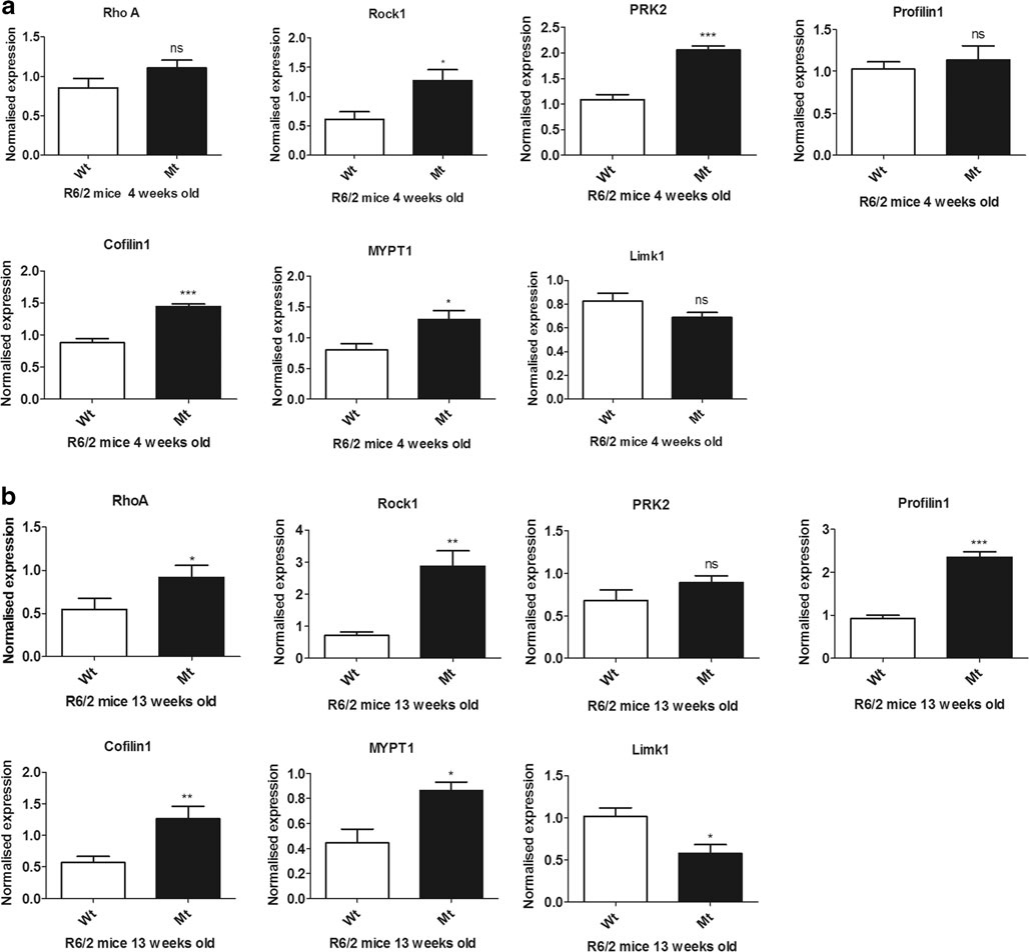
Expression levels of Rho kinase pathway genes in striatum of R6/2 mouse model of HD in early (4 weeks) and late stage of HD (13 weeks) quantified using qPCR. **a** By 4 weeks (early symptomatic), there is a significant increase in the mRNA levels of Rock1, Prk2, Cofilin1, and MYPT1 in transgenic R6/2 mice (*n* = 3–5) compared with wild type mice (*n* = 3–4). No significant difference in mRNA levels of RhoA, Profilin1, and Limk1. **b** At 13 weeks (late symptomatic), there are significant increases in RhoA, Rock1, Profilin1, Cofilin1, and Mypt1 mRNA, whereas Limk1 mRNA was decreased significantly in transgenic R6/2 mice (*n* = 3–4) compared with wild-type mice (*n* = 3–5). There was no alteration in Prk2 mRNA levels at 13 weeks. Expression was normalized with β-actin. Data were expressed as mean ± S.E.M. *p ˂ 0.05, compared with the control

To further investigate alterations in Rho kinase pathway genes in HD, we used qPCR to quantify the mRNA levels of the Rho pathway genes in the striatum of R6/2 HD mice in early and late stages of disease progression (4 and 13 weeks) [13]. At 4 weeks, there was already a significant increase in mRNA levels of Rock1 (2.08-fold, *p* = 0.0125), Prk2 (1.89-fold, *p* = 0.0002), Cofilin1 (1.64-fold, *p* = 0.0002), and MYPT1 (1.62-fold, *p* = 0.0242) and no significant difference in RhoA (*p* = 0.0823), Profilin1 (*p* = 0.3022), or Limk1 (*p* = 0.0815) as shown in Fig. 2a. In late disease, there were significant increases in mRNA levels of RhoA (1.68-fold, *p* = 0.0448), Rock1 (4.07-fold, *p* = 0.0023), Profilin1 (2.55-fold, *p* = 0.0001), Cofilin1 (2.21-fold, *p* = 0.0089), and Mypt1 (1.95-fold, *p* = 0.0153), whereas Limk1 mRNA was decreased significantly with a fold change of 0.57 (*p* = 0.0124) in transgenic R6/2 mice compared with wild-type mice as shown in Fig. 2b. There was no significant difference in Prk2 mRNA levels in the late stage (*p* = 0.1288). We observed increased fold changes in mRNA levels for RhoA (0.39-fold), Rock1 (1.99-fold), Profilin1 (1.44-fold), Cofilin1 (0.57-fold), and Mypt1 (0.33-fold) comparing older with younger transgenic R6/2 mice. mRNA levels of Prk2 and Limk1 mRNA levels were decreased with fold change of 0.57 and 0.26, respectively.

### ROCK1, Profilin1, and Cofilin1 Protein Levels in the Human Postmortem Brain Tissues

**Fig. 3:**
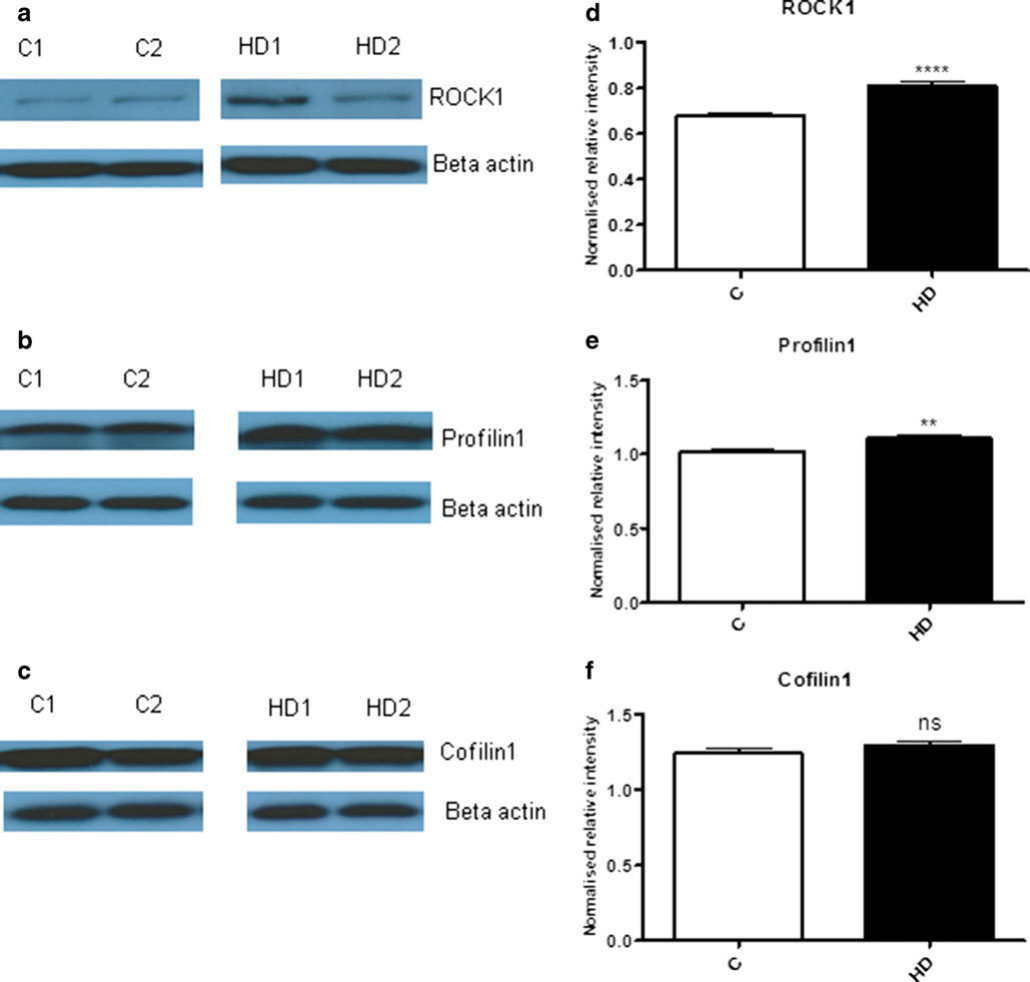
ROCK1, Profilin1, and Cofilin1 protein levels in human postmortem brain tissues. Western blot analysis of protein levels of **a** ROCK1, **b** Profilin1, and **c** Cofilin1 and their densitometric quantifications are shown in (*d*), (*e*), and (*f)* respectively. There is a significant increase in the protein levels of ROCK1 and Profilin1 compared in HD postmortem brain tissues (*n* = 14) compared to controls (*n* = 14). A representative Western blot is shown. There was no significant alteration in Cofilin1 levels. Protein levels were normalized with β-actin. Data were expressed as mean ± S.E.M. *p ˂ 0.05, compared with the control

To determine whether the identified mRNA alterations are translated to altered protein regulation in human HD brain tissue, we have quantified the protein levels of ROCK1, Profilin1 and Cofilin1 using Western blot analysis. Small but significant increases in ROCK1 (p < 0.0001) and Profilin (p = 0.0044) were found in HD compared to controls as shown in Fig. 3d, e and there was no significant change in cofilin1 protein levels (Fig. 3f).

### Rock1 Protein Levels in the Late Stage of R6/2 Mouse Brain Tissue

**Fig. 4:**
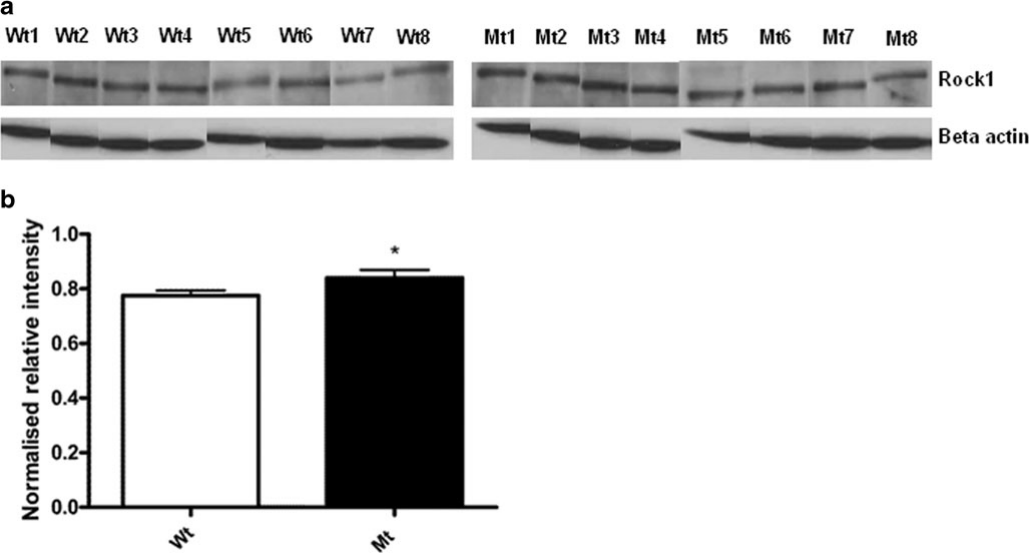
Western blot analysis of Rock1 protein levels in late stage transgenic R6/2 mouse brain. **a** Western blot showing Rock1 protein levels at 13 weeks of age. **b** Densitometric quantification of Rock1 protein levels from wild type (*n* = 8) and mutant type (*n* = 8) in each group showed increased levels of Rock1 protein in mutant type compared to wild type. Data were normalized with β-actin levels. The results were considered significant when *p* ˂ 0.05

To further validate the changes observed in the mRNA levels of Rho kinase pathway genes, we examined Rock1 protein in the brain in 13-week-old R6/2 mice and littermate controls. Western blot analysis revealed there is a significant increase in Rock1 protein levels in the transgenic R6/2 mice compared to the wild-type mice as shown in (Fig. 4a) and by its densitometric quantification as shown in Fig. 4b.

## Discussion

**Fig. 5:**
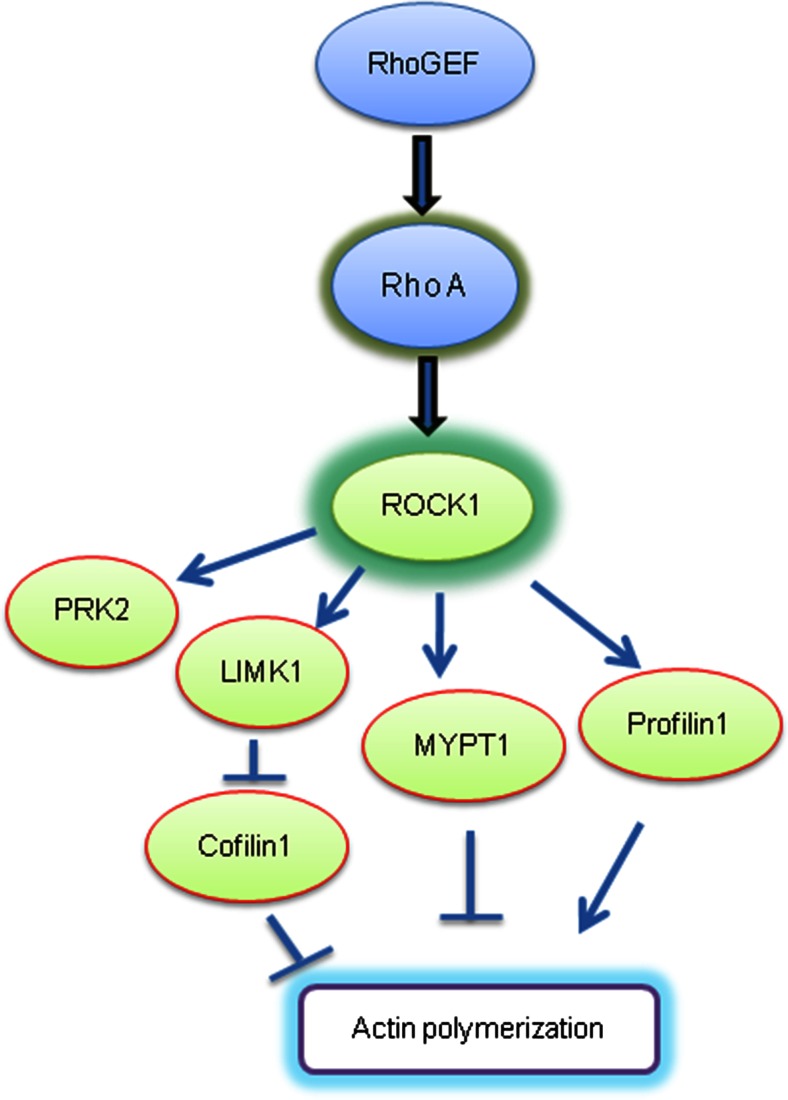
Molecular signaling partners of ROCK1. This map illustrates the Rho kinase signaling pathway. ROCK1 is the key effector and its downstream targets are all important regulators of a variety of cellular functions which control cell morphology, cell migration, cytoskeletal reorganization, cell differentiation, and cell death. Each of these gene targets are altered in HD

Rho kinases regulate cytoskeletal structural proteins and play a major role in regulating cell growth, cell structure and differentiation, migration, energy conversion, and cell death [14–17]. A potential role for the Rho kinase pathway in the pathogenesis of HD has also emerged along with its potential as a target for disease modification. Here, we report a panel of Rho kinase pathway genes (Fig. 5), which are coordinately upregulated in leukocytes and in the brain in HD subjects. In R6/2 transgenic mice, we show this same upregulation in the brain and that it is heightened in later stages of disease in which additional genes are also upregulated, suggesting these changes may correlate with progression. These findings replicate by QPCR and extend our earlier findings from genome-wide microarray studies that two Rho kinase pathway genes, ROCK1 [10] and Cofilin1 [11] are increased in HD leukocytes and suggest that some of these genes could be a useful source of peripheral markers of HD progression or of pharmacodynamic responses to treatments targeting it.

The Rho/Rock pathway primarily regulates actin cytoskeletal rearrangements through activation of downstream substrates such as MYPT1, LIM kinases and triggers caspase-mediated apoptotic cell death via plasma membrane blebbing [15, 17–19]. Rock can be constitutively activated by proteolytic cleavage of the inhibitory carboxyl terminal domain. Rock1 is cleaved by caspase 3 at the cleavage site DETD1113 during apoptotic condition [18]. Caspase 1 and caspase 3 are transcriptionally upregulated and activated in transgenic HD mice [20], suggesting that caspase 3 could constitutively activate Rock1 in HD. Previous studies in HD cell culture models have demonstrated that Rock1 and protein kinase C-related protein kinase (PRK2) are involved in mediating mtHtt aggregate formation and Rock inhibition decreases aggregation [9]. Considering these factors together, the upregulation of the Rho/Rock pathway may mediate phosphorylation of its downstream targets which signal toward apoptotic cell death. However, it is unclear how Rho/Rock pathway is transcriptionally regulated in HD pathogenesis.

In HD leukocytes, Rock1 and Profilin1 had fold changes of 1.32 and 1.47, respectively, while cofilin1 had the highest fold change of 1.63, sufficient differences to serve as potential peripheral markers of HD clinically. Rho kinase pathway gene upregulation in HD postmortem brain tissues paralleled those in the blood and had even higher fold changes (up to 2.58 for Rock1 and 3.58 for Profilin1) suggesting that mtHtt affects the Rho kinase pathway similarly in the brain and blood. In addition to increased mRNA levels of ROCK1 and Profilin1, we observed modest, but significant upregulation of the protein levels in human postmortem brain. These genes could thus usefully mark target engagement by potential treatments in the blood from research subjects and in mouse tissue in translational studies. Future studies with larger cross-sectional and longitudinal patient sample sets are necessary to examine whether the expression of these genes, either individually or as a panel, can mark the HD prodrome, clinical onset, or disease progression. Additional studies using R6/2 HD mice treated with Rho kinase inhibitors could help validate these genes as pharmacodynamic markers.

In conclusion, the present study shows that the presence of mutant huntingtin causes increased expression levels of Rho/Rock kinase pathway genes in HD leukocytes, postmortem brain tissues, and in R6/2 HD mice. However, more studies are necessary to clearly understand the precise molecular mechanism of upregulation of this pathway in HD pathogenesis, and to further validate its potential for providing novel biomarkers and disease-modifying therapeutic targets for HD.
